# Effect of Sample Size and Crystal Orientation on the Fatigue Behaviour of Single Crystalline Microbeams

**DOI:** 10.3390/ma13030741

**Published:** 2020-02-06

**Authors:** Jorge Rafael Velayarce, Christian Motz

**Affiliations:** Institute of Material Science and Methods, Saarland University, 66123 Saarbrücken, Germany; motz@matsci.uni-sb.de

**Keywords:** fatigue, PSBs-like structures, kink bands, dislocation dipole

## Abstract

Beam deflection experiments were used to systematically examine size effects on the low cyclic fatigue (LCF) deformation behaviour of micro-sized bending beams of copper (Cu) single crystals oriented for single slip, critical and coplanar double slip. We present cyclic hardening curves and fatigue surface roughness, as well as dislocations structures of the micro-sized beams with sizes between 1 and 15 µm. A clear crystal orientation and size effect on the cyclic hardening curves, surface roughness, and the dislocation microstructures were observed. Based on the experimental results, the fatigue damage in single slip orientations clearly decreased with decreasing the sample size, however, below a critical size regime, the surface damage suddenly increases. Additionally, samples with sizes larger than 5 µm clearly revealed, besides PSBs-like structures, the emergence of kink bands leading to larger surface roughness in comparison to the smaller ones. Fatigue surface damages in microcrystals oriented for critical double slip became more prevalent compared to single slip orientations. Quantitatively, the correlation of the fatigue surface damage was also demonstrated with the formation of PSBs-like structures.

## 1. Introduction

Fatigue is one of the most important failure mechanisms in engineering structures [1]. Numerous experimental works [1,2] have shown that the origin of (macro) fatigue failure is related to dislocation structures (cells, labyrinths, veins, and persistent slip bands (PSBs)) which are dependent on the crystal orientation [1,3,4]. It has also been demonstrated that repetitive loading (cyclic accumulation of slip irreversibilities) in bulk materials (single and polycrystals) causes surface fatigue damage in the form of slip bands/PSBs [5,6,7,8,9] and/or deformation bands (DB I, DB II, and DB III) [10,11,12]. PSBs normally have the form of ribbon- and tongue-like extrusions/intrusions [6,13,14,15] and lead to surface roughening and eventually to a fatigue crack. Exceedingly collective and cooperative dislocation effects are responsible for the PSBs, where the mutual annihilation of edge dislocation dipoles, production of vacancies, cross slip of screw dislocations, and random gliding result in slip localization within PSBs [5,6,8,16]. Another, less common type of strain localization is kink band formation that has been observed in bulk single crystals deformed under unidirectional and fatigue deformation [5,12,17,18]. This kink band deformation, which is approximately perpendicular to the active slip plane, is crucial to understand the fatigue mechanisms in ductile bulk materials.

Due to the accelerating pace of miniaturization in many technological areas, the interest in the micromechanical evaluation of small-scaled samples/components (the “smaller is stronger” phenomenon) has increased continuously [19,20,21,22,23,24]. Particularly in the nano- and microelectronic industry where microcomponents are unavoidably subjected to cyclic loading which consequently can cause fatigue damage. Despite the great fatigue testing challenges and the lack of a proper understanding of fatigue behaviour of small scaled materials, some studies have demonstrated that single [25,26,27] and polycrystalline [28,29,30] microcrystals with sizes located at the same size regime as dislocation structures or below [31] showed markedly different fatigue properties compared to bulk materials. For example, based on [28,29] the morphologies of the fatigue-induced extrusions, cracks, and dislocation structures of thin films-substrates composite, developed by Kraft [30], are basically controlled by both the film thickness and grain size. It has been observed that the fatigue surface damage in LCF and the high cycle fatigue regime decreases with film thickness and grain size and that the failure stress amplitude increases for films thinner than 0.6 µm [28,29,32]. This fatigue behaviour change, from extrusions to interface-mediated damage, is attributed to the confinement on the dislocation (at least in one dimension) motion and multiplication affecting dislocation arrangements. Therefore, characteristic dislocation microstructures disappear in films smaller than 3 µm and only individual dislocations are observed in films < 1 µm [29]. To avoid microstructure or substrate effects of the mentioned techniques, Kiener et al. [25] proposed a new technique based on the microbeam deflection, where geometrically necessary dislocations (GNDs) due to larger strain gradients have important consequences on the plastic deformation of microcrystals during load path changes. In combination with discrete dislocation dynamics (DDD) simulations, it was observed that GNDs in the pile-ups go hand in hand with the Bauschinger effect, which increases with the strain amplitude and decreasing sample size. A detailed study of the Bauschinger effect on the plasticity of microbeams was carried out by Demir et al. [27] and Kirchlechner et al. [26]. Based on these studies, the evolved GNDs are clearly associated with the Bauschinger effect, which leads to a reversibility of plastic deformation. However, the sample size effect has been investigated only up to 3 [27], 22 [26], and 100 loading cycles [25]. The cyclic deformation behavior of microbeams under cyclic loading conditions with strain gradients for higher cycle numbers has not yet been investigated. Additionally, in order to better understand the underlying mechanisms of fatigue behaviour of microsamples with dimensions located above and in the same size regime as that of, e.g., ladder-like structures and their correlation with evolved dislocation structures, this needs to be investigated at larger loading cycles.

Therefore, this work systematically investigates the fatigue surface damage and dislocation microstructures in single crystals, as well as their crystal orientation effect within a larger size regime (from 15 down to 1 µm) in the LCF regime (up to 10^4^ cycles).

## 2. Experiments

### 2.1. Sample Preparation and Characterization

**Figure 1:**
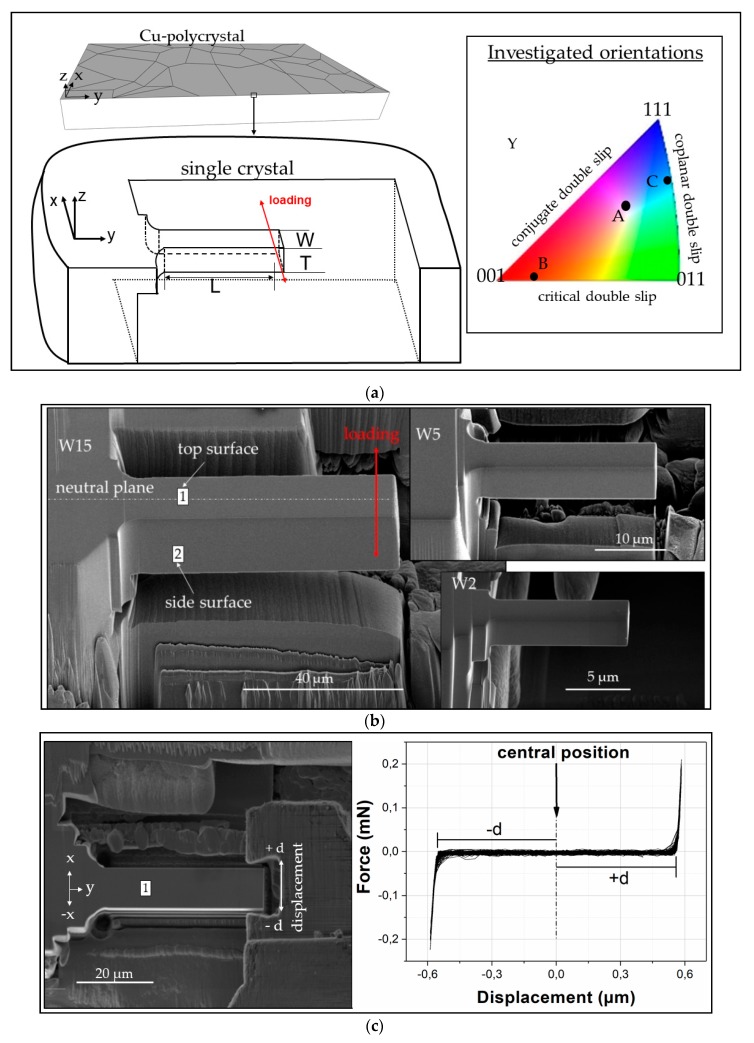
(**a**) Schematic representation of the used microbeam geometry with a square cross-section and an aspect ratio 4:1 (W:L), where the loading direction is parallel to the x direction. The crystallographic orientations used for single slip (SS), critical double slip (DS), and coplanar double slip (DS) are marked with A, B, and C, respectively in the standard stereographic triangle. The orientations in the triangle indicate the direction parallel to the neutral plane of the beam. (**b**) Representative SEM images of three microbeams with a width of 15, 5, and 2 µm before fatigue testing and (**c**) the end alignment of the gripper and beam, as well as the elastic force-displacement curve (see text for details).

The used material for the fabrication of microbeams samples was polycrystalline Cu with a purity of 99.99%. A set of plates with dimensions of approximately 2.5 × 5 × 15 mm (Figure 1) was prepared. These macrosamples were ground with grit-paper and then heat-treated in vacuum at 1100 °C for three days to grow the grain size to an average of about 1 mm. In a next step, the samples were ground again and carefully polished with diamond suspension. Subsequently, we used phosphoric acid (electrolyte D2) for the electrolytic polishing of the sample to remove any oxide layers, as well as other surface contamination or deformations, which would affect and degrade the electron backscattered patterns. The determination of the crystal orientations was achieved by electron backscattered diffraction (EBSD) in a Carl Zeiss SIGMA series scanning electron microscope (SEM, Oberkochen, Germany). To systematically investigate the effect of sample size on the cyclic deformation behaviour of microsamples, we focused our study on Cu single crystals oriented for single slip (SS). The SS phenomena are of great importance because in, e.g., high cyclic fatigue of polycrystals the greatest fraction of deformation is carried out by the softest grains (dominated by SS). In addition to SS orientations, double and multiple slip phenomena are as well very important to provide insights into the fatigue behaviour of polycrystalline materials [9]. Therefore, we selected two additional crystal orientations to observe the crystal orientation effect on the damage accumulation in microsamples. In the standard stereographic triangle in Figure 1a we marked the three orientations (A, B, and C), which are parallel to the beam neutral plane (beam axis orientation): (i) For single slip (SS) deformation, the orientation [-63-2] is located inside the stereographic triangle, (ii) for critical double slip deformation the orientation [0-51] is near the 001/011 side, and (iii) for coplanar double slip deformation the orientation [5-53] is near the 011/111 side of the stereographic triangle.

### 2.2. Microsample Fabrication

The micrometre-sized beams were fabricated at the sample edge using the focused ion beam (FIB) technique with a FEI Versa 3D (Hillsboro, OR, USA) along the y-direction of the sample (see Figure 1a). The microbeams were fabricated using currents of 15 nA for rough cutting and 100–500 pA at 30 kV for fine polishing. Low currents are important to minimize Ga-damaging, material redeposition, and to achieve a well-defined beam geometry (Figure 1b). We investigated beams with a thickness between approximately 1 and 15 µm to investigate the role of thickness in the development of dislocation structures (surface damage evolution) during cyclic deformation. This wide size regime covers a sample size range where the strength of single crystals is strongly influenced by the extrinsic sample volume [19] and where the GNDs and dislocation pile-ups have a dominant effect resulting in an enhanced Bauschinger effect [21,24,25,27]. Additionally, reducing the sample dimensions far above and below the material length scale discussed in [33,34] one can clearly observe the effect of strain gradients, e.g., on the deformation-induced internal stresses and lattice plane misorientation, as pointed out by [35].

The aspect ratio (L:W), the ratio between the moment arm with the length (L) and the beam width (W), was approximately 4:1 for all beam sizes. To reduce stress concentrations at the beam corners, where the moment is maximum, all beams were fabricated with radius r (see Figure 1a).

### 2.3. Mechanical Testing in a SEM

The fatigue tests were carried out in high vacuum at room temperature in a SEM equipped with an in situ nanoindenter (UNAT-SEM II, ASMEC, Dresden, Germany). The microbeams were fatigued under full load reversal (Figure 1c). The used grippers were fabricated from polycrystalline tungsten carbide (WC) because of its good mechanical characteristics [36]. Figure 1c shows the end alignment of the gripper and the beam. Due to the gap between the beam and the gripper, it is crucial to find the central position in order to have the same deformation in +x and −x direction. Therefore, prior to the fatigue deformation, using a sine-form function under displacement control, few cycles were performed elastically to adjust the distance d (see force-displacement curve in Figure 1c). After the last procedure, we increased the displacement amplitude.

All microbeams were deformed cyclically up to 10^4^ cycles with a frequency of 1 Hz, a stress ratio R = −1, and a plastic strain $\Delta \varepsilon_{p}$ of ~10^−4^ to ~10^−2^. The fatigue damage morphologies of all microbeams were analyzed in the SEM after 10^4^ cycles. After the SEM imaging, we polished the fatigue surface roughness to characterize the distribution of dislocation structures (GND density) with the EBSD technique and with the back-scattered electron (BSE) detector. The cleaning of the surface roughening was performed using ion beam currents of 1 down to 0.1 nA at 30 and 5 kV.

## 3. Results

The investigated beam orientations and the fatigue testing conditions are summarized in Table 1 and the corresponding Schmid factors for all slip systems are in Table 2. In this work, the following comparisons have been made: (i) The cyclic stress response, (ii) the surface damage morphology, and (iii) the distribution of dislocation structures as a function of sample size and crystal orientation.

### 3.1. Cyclic Stress-Strain Curves of Single Crystal Beams

**Figure 2:**
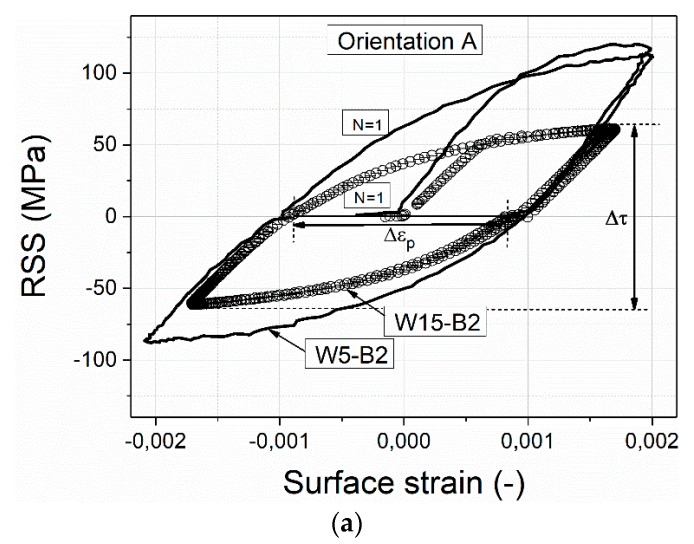

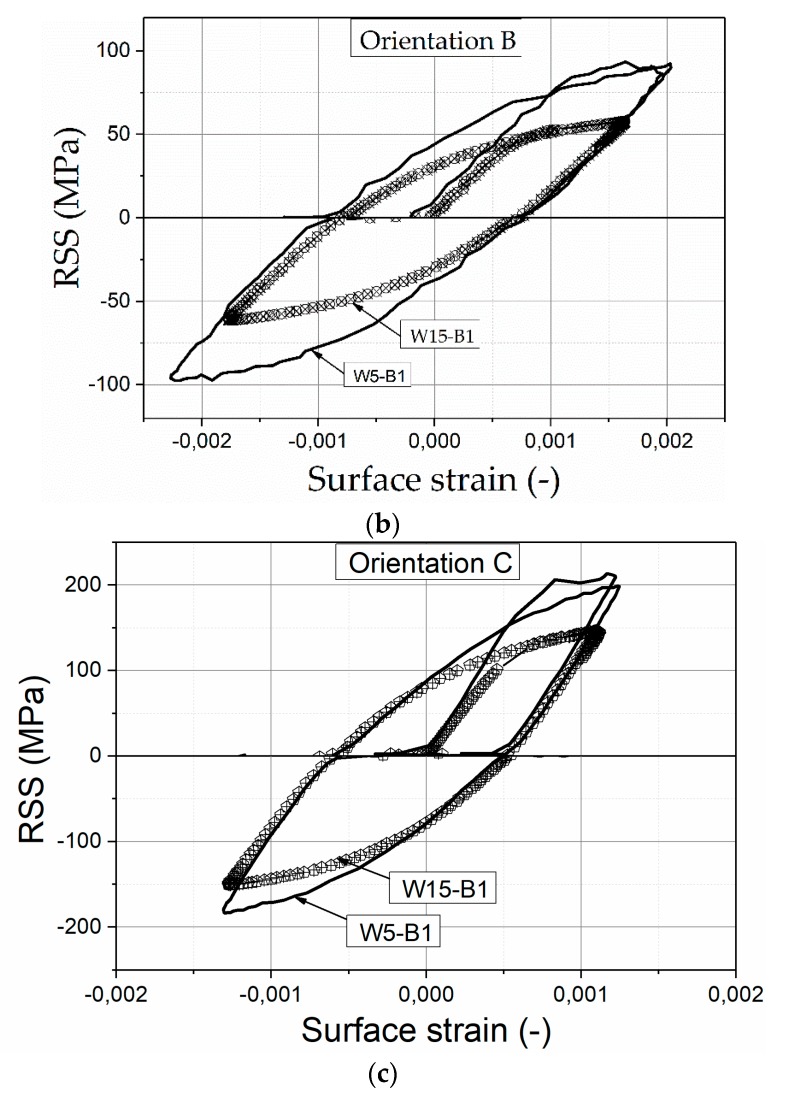
Representative cyclic stress-strain curves for beams with a width of 15 and 5 µm (W15 and W5) oriented for (**a**) single slip, (**b**) critical double slip, and (**c**) coplanar double slip.

Figure 2 shows typical stress-strain hysteresis loops during cyclic deformation of the tested microbeams. Figure 2 displays only the first hysteresis loops of a large (W15) and medium-sized (W5) beam with the orientation A, B, and C. Independent of the crystal orientation, the overall resolved shear stress (RSS) of the smaller beams is higher than the larger ones (observed also under monotonic loading) cycled with an approximately constant strain amplitude. In all three crystal orientations, we can see clear differences in the RSS during the first loading cycle of the W5 beams, while the W15 beams do not show this feature and their shape of the hysteresis loops are similar to the loops observed in macrosamples [5]. Another feature is the increasing Bauschinger effect (BE) (larger yielding stress in the forward direction in comparison to the reverse direction) as the sample size decreases as expected in [25,27].

### 3.2. Cyclic Stress Curves of Single Crystal Beams with Different Crystal Orientations after 10^4^ Cycles

**Figure 3:**
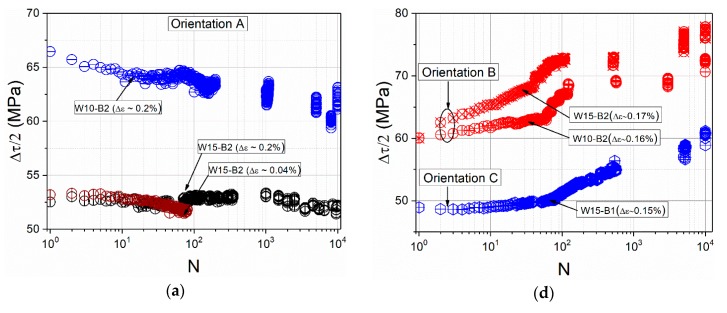

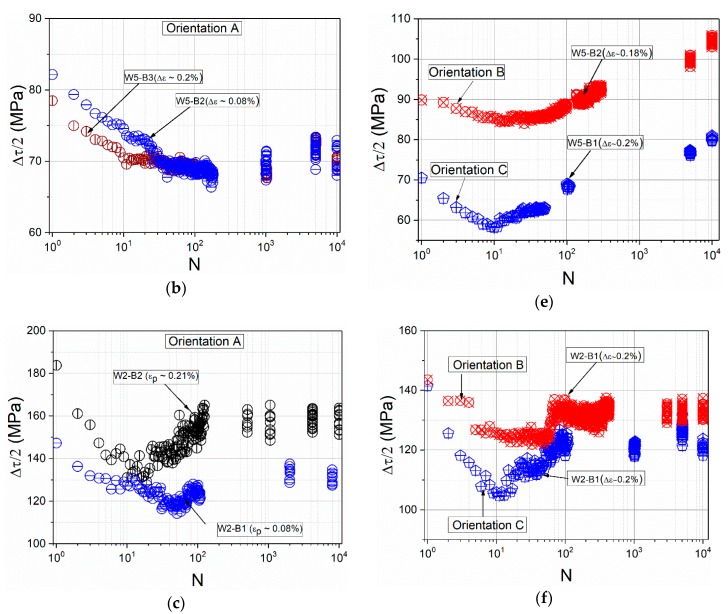
Cyclic stress curves of beams with the orientation A, B, and C: In (**a**–**c**) are results of the larger (15 and 10 µm), medium (5 µm), and smaller (2 µm) beams, respectively, with the A orientation and in (**d**–**f**) the results of the larger, medium, and smaller beams, respectively, with the B and C orientation.

In order to make a direct connection between the cyclic stress response and the cyclic damage morphology, as well as the dislocation structures, we used the cyclic hardening curves of representative beams with the orientations A, B, and C. Figure 3 shows the cyclic hardening curves of microbeams in terms of the mean RSS Δτ/2. Figure 3a–c show the cyclic stress curves of the beams with orientation A. Figure 3d–f display the results corresponding to the beams with B and C orientations.

Figure 3a–c clearly show the sample size effect on the cyclic stress response of beams with SS orientation loaded with a similar plastic strain $\Delta \varepsilon_{p}$. Compared to the cyclic hardening curves of bulk single crystals [5,6,10,37], which show cyclic hardening after few cycles depending on the $\Delta \varepsilon_{p}$, the W10 and W15 microbeams oriented for SS do not exhibit hardening but rather a cyclic softening at higher cycle numbers (softening clearer in W10 than in W15).

The absence of cyclic hardening in single crystal beams was also observed by Kiener et al. [25], where the fatigue tests were performed with similar conditions but only up to 100 cycles. This cyclic softening during the first loading cycles happened for nearly all microbeam sizes and it becomes more pronounced as the sample size is reduced. This can be clearly observed independent from the crystal orientation in Figure 3b,c,e,f. Interestingly, the results that have attracted attention are from the smaller-sized beams whose cyclic stress curves exhibit three clearly marked regions, where different mesoscopic dislocation-based mechanisms are responsible for the cyclic softening, hardening, and saturation-like state. All W2 beams with A, B, and C orientation clearly showed a marked softening during the approximately first 20 cycles whereby the cyclic stress of beams with A and C orientation drops lower compared to the B orientation. After approximately 20 cycles corresponding to an accumulated plastic strain $\varepsilon_{p,cum}$ of 0.08 ($\varepsilon_{p,cum}=2N\Delta \varepsilon_{p}$, where N is the number of cycles) the beam with SS orientation showed a hardening with increasing cycle number and suddenly after about 100 cycles ($\varepsilon_{p,cum}=0.4$) entered into a saturation-like state, as well as the beams with B and C orientation.

In the case of the larger beams with the orientation B and C we clearly observed cyclic hardening (Figure 3d,e), whereby the microbeams with orientation B show a pronounced cyclic hardening and the microbeams with orientation C show a clear hardening only starting as of approximately 60 cycles.

In the case of the beams with a thickness of 5 µm (Figure 3e), the RSS decreases during the first cycles (approximately between N = 1 and 30 cycles) independent of the crystal orientation. After a certain cycle number, the RSS increases continuously as also observed in the larger beams (Figure 3d), while the RSS of the beams oriented for SS (Figure 3b) remains relatively constant even up to 10^4^ cycles.

Table 3 summarizes a comparison between the three orientations in terms of initial RSS, minimum RSS after few cycles, saturation-like RSS, and characteristic lengths of dislocation structures in the microsamples and in bulk material.

### 3.3. Damage Morphology of Single Crystal Beams after N = 10^4^ Cycles

**Figure 4:**
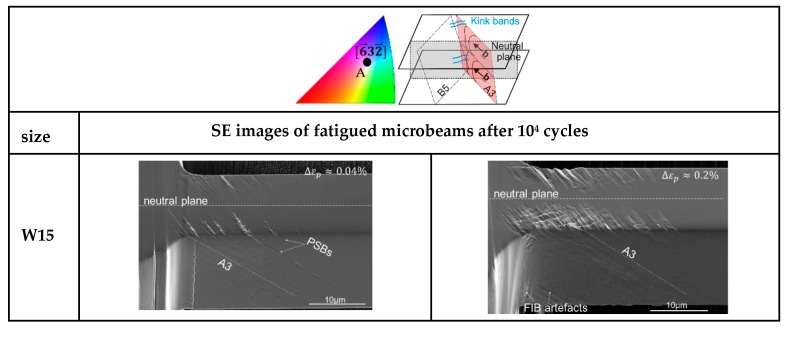

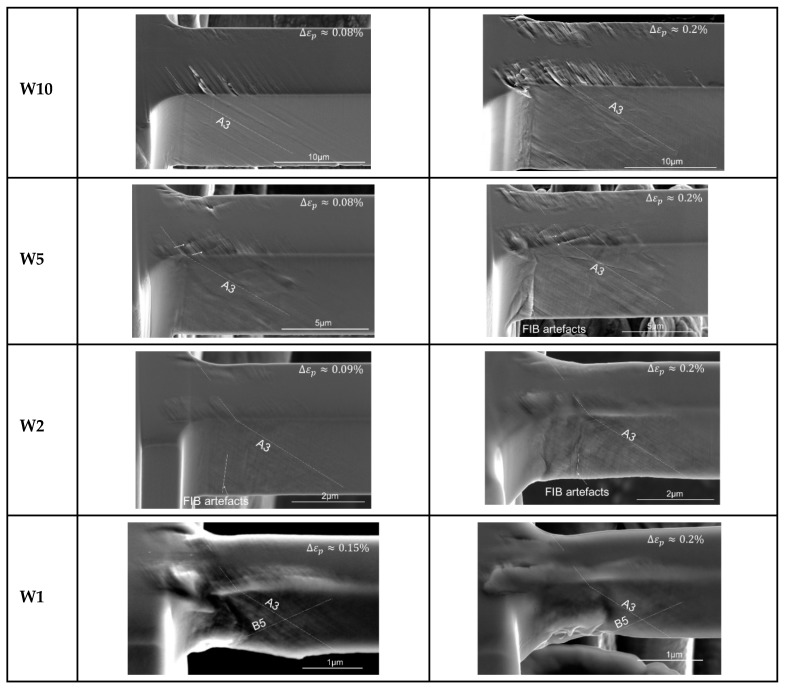
Evolution of the surface roughness of microbeams oriented for single slip with different sizes (from 15 to 1 µm (W15 to W1)). Left column: Small strain amplitude and right column: Larger strain amplitude $\Delta \varepsilon_{p}$ (see values on the respective beams). Orientation and the sketched glide systems A3 and B5 with the largest Schmid factor are on the first row for more clarity.

Mughrabi [5] showed that the applied strain amplitude has a clear influence on several features such as the appearance, broadening, and number of PSBs, as well as on the dislocation response and structures. Therefore, we systematically investigated the development of the surface roughening as a function of the sample size at the low amplitude fatigue region (Region B in cyclic stress-strain curve [5]). Figure 4 summarizes the surface slip morphology of microbeams oriented for SS, with thickness ranging from 15 down to 1 µm after 10^4^ cycles fatigued with small and higher $\Delta \varepsilon_{p}$. The higher magnification SEM images are close to the fixed end region, where the largest strain prevails. The SE images in Figure 4 clearly reflect the influence of the strain gradient, the imposed strain amplitude of cycling, and sample size on the surface damage. As we clearly see in Figure 4, plastic deformation emerges at the beam surface and ends around the neutral plane indicating the existence of a strain gradient. Figure 4 also shows that the PSBs-like slip bands are mostly concentrated on the active (primary) slip system A3 in the W15 microbeams while in the W1 beams besides A3 the slip system B5 is also activated. Individual slip bands with a thickness of approximately 1.5 µm of the W15 beam deformed at $\Delta \varepsilon_{p}\sim 0.04\%$ are similar to PSBs with a tongue-like feature as observed in bulk materials [13,38]. However, increasing the strain amplitude, the cyclic strain does not concentrate on individual slip bands but distributes almost uniformly at the beam surface. In addition, we can also observe deformation bands in the form of micro kink-like bands (KBs) (see sketch and arrows in Figure 4) which arise approximately perpendicular to the glide system A3 analogous to the DBII in macrosamples. Qualitatively, Figure 4 clearly reflects that the fatigue surface roughening decreases gradually when reducing the sample size (both for lower and higher $\Delta \varepsilon_{p}$) as expected in the literature [25,27]. This surface roughening decrease can be observed most clearly when comparing the W15 beam fatigued with $\Delta \varepsilon_{p}$ ≈ 0.04% and the W2 beam fatigued with a higher $\Delta \varepsilon_{p}$ ≈ 0.09%.

However, there is a size regime (≤1 µm) where this slip irreversibility clearly increases again. In addition to the decreasing of the surface roughness with the sample size, we also see that the emergence and the distance between the KBs clearly decrease (from ~1.7 µm in W15 to ~0.7 µm in W5) and then around the sample size W2 these KBs are no longer visible at both strain amplitudes. While in the smallest microbeam (W1) the fatigue damage increases, it seems that the slip lines are closer to each other and homogeneous and that some faint intrusions form at the surface (not along individual slip bands such as in larger beams).

**Figure 5:**
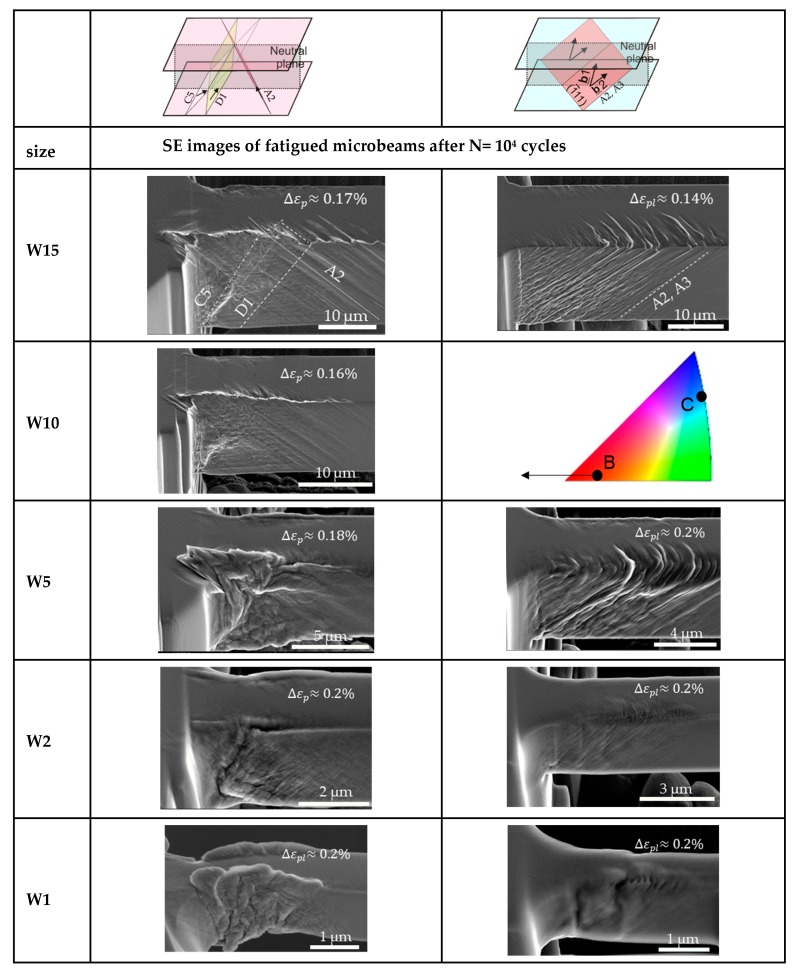
Evolution of the surface roughness of microbeams with different sizes (from 15 to 1 µm) after 10^4^ cycles. Left column: Beams with the orientation B (critical double slip); right column: Beams with the orientation C (coplanar double slip). Sketched glide systems A2, D1, and C5 (orientation B), and A2 and A3 (orientation C) with the largest Schmid factor are on the first row for more clarity.

As stated above, in order to investigate the crystal orientation effect on the fatigue damage evolution in small samples and to qualitatively compare the results obtained in the single slip, we additionally present the surface morphologies of the microbeams with the orientation B and C in Figure 5. It is evident that the surface morphology, as well as the cyclic hardening curves of the microbeams are strongly influenced by the crystallographic orientation. For example, beams with orientation B show qualitatively higher fatigue damage characteristics where the slip system A2 and D1 are highly visible (see schematic illustration for more details). The fatigue surface roughness slightly deviates from the beams with orientation C and significantly from the beams oriented for SS. The fatigue damage evolution decreasing the sample size in orientation C shows also an almost similar tendency as the beams with SS orientation. While beams oriented for critical double slip show a hardly discernible influence/tendency of the surface morphology with decreasing sample size until W2. However, below W2 microbeams reveal a smeared dislocation flux at the surface causing a complex and very different surface roughening compared to the larger samples.

### 3.4. Dislocation Structures after 10^4^ Cycles

**Figure 6:**
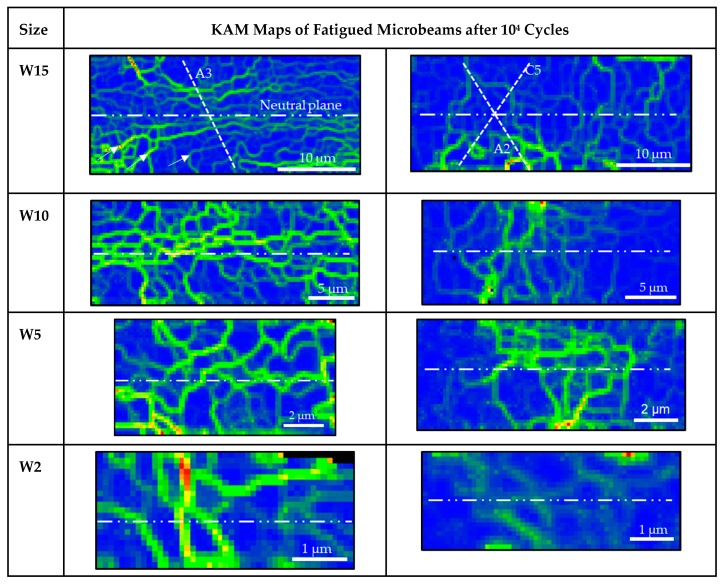

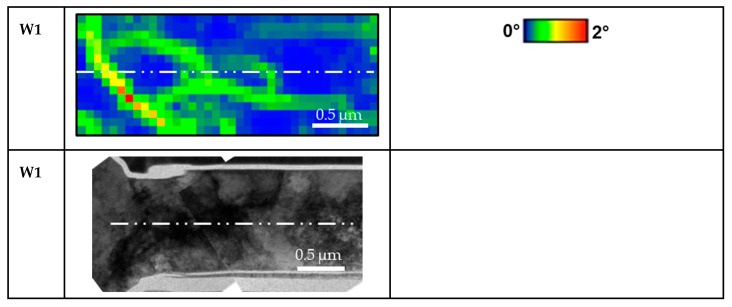
Kernel average misorientation (KAM) maps showing the evolution of the dislocation structures of the microbeams with the orientation A (left column) and orientation B (right column): The KAM maps on the left side are of the beams in Figure 4 deformed with Δε_p_ ≈ 0.2% and the KAM maps on the right side of the microbeams with orientation B in Figure 5 (these KAM images were taken on the top surface of the beams (surface 1 in Figure 1b)). The TEM image of a beam with the width of 1 µm on the left side showing diffuse structures was used for a qualitative comparison with the KAM image of beam size W1.

The dislocation structures of the microbeams with the crystal orientation A and B after 10^4^ cycles are summarized in Figure 6. The EBSD maps were taken on the surface 1 (top side) of the microbeams (see Figure 1c). The dislocation patterns on the left side (orientation A) correspond to the beams in Figure 4, deformed at larger strain amplitudes and the structures on the right side (orientation B) to the beams in Figure 5. The EBSD method can also be applied to analyze the mesoscopic dislocation structures and the local lattice misorientation. Thus, the kernel average misorientation (KAM) maps (Figure 6) can be used as an approximation for the density of GNDs [27]. Similar to the surface roughness in Figure 4, the KAM maps clearly show a strain gradient, sample size, and crystal orientation effect on the dislocation structures. For example, in the SS orientation, the dislocation structures clearly vary from the surface to the neutral plane direction; most notably in the larger beams. The KAM maps also clearly reveal some dislocation walls (see arrows) which are distributed approximately perpendicular to the slip system A3 showing a correlation to the KBs. We can also observe continuously a decrease of the dislocation structures frequency reducing the sample size. Additionally, we can observe that the dislocation structures continuously change from well-defined dislocation walls/cells to a diffuse dislocation arrangement in the smallest beams W1 (KAM and TEM image). The TEM measurement was used to have a deeper insight into the dislocation arrangement in a 1 µm microbeam after 1000 cycles deformed with a $\Delta \varepsilon_{p}\sim 1\%$. This TEM image clearly shows diffuse dislocation arrangements despite the high number of cycles. In comparison to the SS orientation, orientation B shows more complex dislocation structures. We can also recognize that the beam size W2 only shows diffuse tangled dislocations and no cell-like structures.

## 4. Discussion

The observed experimental results demonstrated a length-scale influenced cyclic damage, as well as a clear crystal orientation influence. In the following, we will discuss the length scale effect on the surface roughening, the collective dislocation activity and the correlation to cyclic hardening, as well as the crystal orientation influence on the fatigue damage evolution.

### 4.1. Fatigue of Large Microbeams

Based on several extensive works in bulk fcc metals [2,6,39,40], the accumulation of irreversible plastic microstrain is responsible for the surface roughening and consequently the microcracks initiation. After cyclic hardening, macro single crystals oriented for SS clearly evidence a saturation plateau corresponding to the PSBs formation characterized by the saturation shear stress, $\tau_{S}$, in the range 27–30 MPa. PSBs normally form parallel to primary slip planes where several collective dislocations move simultaneously. Collective dislocation effects are mainly responsible for slip localization particularly in PSBs in which dislocations, after a certain cumulative strain, rearrange into unique dislocation structures in order to minimize the energy of the system [6,16,41]. This means that a certain dislocation density is necessary for the formation of ladder-like structures with a PSB wall spacing range of 1–1.3 µm [3,41]. Thus, the collective interactions of a certain dislocation density in bulk materials contribute to rapid initial cyclic hardening even during the first ten cycles depending on the crystallographic orientation and strain amplitude [42,43].

**Figure 7:**
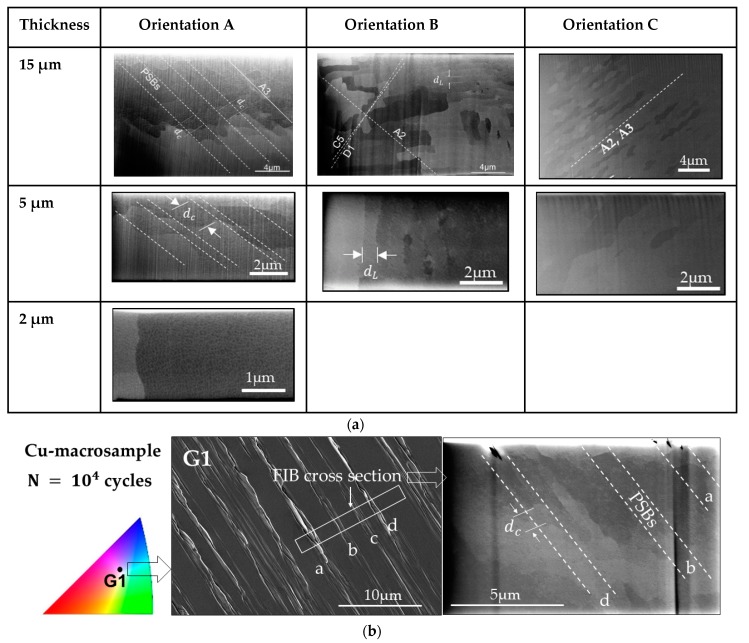
(**a**) Back-scattered electrons (BSE) images with the characteristic dimension of PSB-ladder and labyrinth structures, the PSB wall spacing *d_c_* and labyrinth spacing *d_L_* after 10^4^ cycles of microbeams with the dimension of 15, 5, and 2 µm (W15, W5, and W2) with the orientation A, B, and C (this image was taken on the side surface of the beams (surface 2 in Figure 1b)). (**b**) In addition, a SE image of the tongue-like extrusions/intrusions of a grain oriented for single slip in a polycrystalline Cu macrosample after N = 10^4^ cycles fatigued with a strain amplitude of $\Delta \varepsilon_{pl}\;\approx 0.2\;\%$ and a BSE image of a cross-section with four PSBs (a, b, c, and d) revealing ladder-like structures.

However, the results in Figure 3 for SS orientation do not show a clear cyclic hardening, even in the larger samples (W15), although it was observed that fcc microcrystals (around 20 µm) oriented for SS showed a nearly bulk-like mechanical response under monotonous loading [44]. According to cyclic deformation behaviour in bulk crystals, one would expect cyclic hardening because of the greater accumulation of dislocations in the form of trapped primary dislocation dipoles (veins formation) which normally contribute to a rapid initial cyclic hardening [41]. However, we observed that the largest microbeams (W15) showed a relatively constant shear stress. The very long heat treatment of the Cu samples and the local microsample fabrication by the FIB method lead to the fact that microsamples can be almost perfect single crystals, whereas this is not the case in bulk single crystals. Therefore, the absence of initial cyclic hardening in the W15 and W10 beam with SS orientation is highly probable due to the low existing dislocation sources in the beams, in contrast to bulk single crystals, which from the beginning contain a high initial dislocation density. Furthermore, the neutral plane, which influences the size effect [24], also affect certainly the evolution of dislocation structures and thus to the cycle stress curves. This initial softening behaviour becomes naturally more evident in smaller beams where the stress gradients are higher, and the dislocation sources become scarcer. Figure 7a clearly shows the sample size/neutral plane influence on the dislocation microstructures (in all three orientations), which notably becomes less frequent with decreasing sample size. Furthermore, the BSE images reveal that a characteristic dimension of the dislocation structures, the PSB wall $d_{c}$ and labyrinth spacing $d_{L}$, do not clearly depend on the sample size. Based on the changes in the background contrast between neighbouring channels, we can clearly recognize PSB walls in the beams W15 and W5 but none (or very hard to image) in the smaller ones. The mean PSB wall spacing, $d_{c}$, is about 1.4 ± 0.1 µm and labyrinth spacing, $d_{L}$, is about 0.8 ± 0.1 µm, which are in the same size regime as in macrosamples [41,45]. To have a reference, a polycrystalline Cu macrosample was additionally fatigued with the same loading conditions as the microbeams ($\Delta \varepsilon_{p}$ and N). The surface roughness and PSB structures of a grain oriented for SS are shown in Figure 7b, where we can observe PSB structures with a similar PSB wall spacing $d_{c}$.

**Figure 8:**
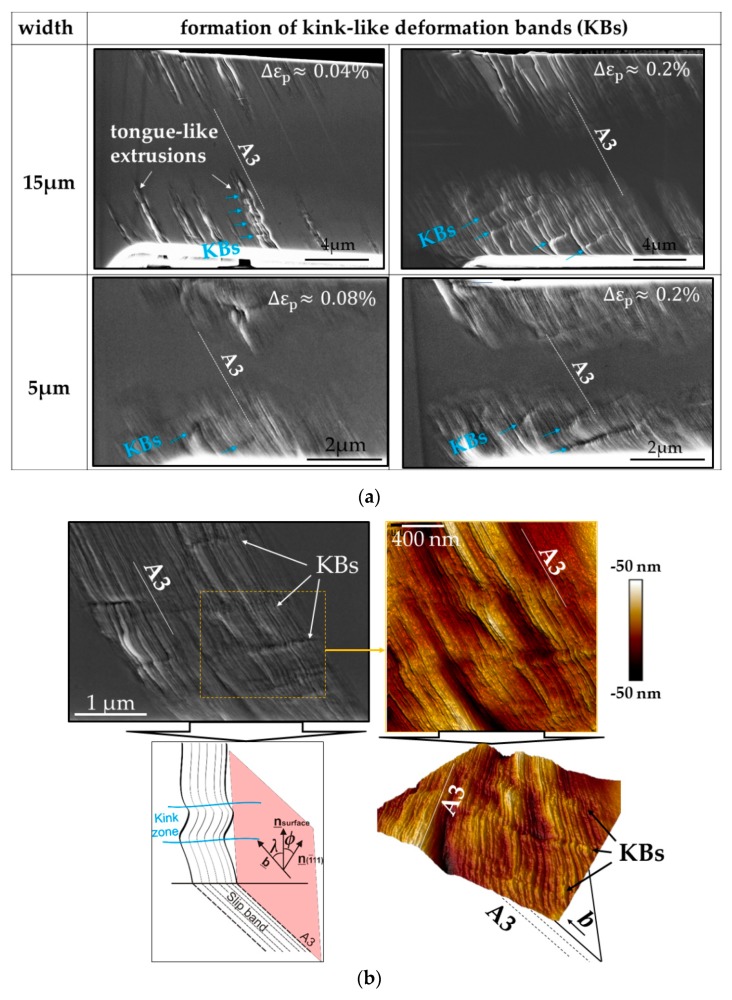
(**a**) SE images of the top surface (surface 1 in Figure 1b) after N = 10^4^ cycles of the 15 and 5 µm beams fatigued with different strain amplitude $\Delta \varepsilon_{p}$; (**b**) SE and three-dimensional atomic force microscope (3D AFM) images of an individual PSB on the top surface of a 15 µm beam, as well as a schematic representation of an individual kink band at emerging slip band.

That means the frequency of dislocation structures, which in the macrosample are more frequent than in the microbeams, clearly correlate with cyclic stress curves and thus with the fatigue damage, which is concentrated on defined primary slip planes. Additionally, the collective action of dislocations in several slip lines A3 during cycling deformation produced slip bands leading to a surface roughness with tongue-like extrusions, as observed in bulk materials [13,38] (see larger beams in Figure 4). More details of the fatigue roughness on the surface of the beams W15 and W5 in Figure 4 are shown in Figure 8a where the periodic array of micro-KBs is clearer. Figure 8a also shows the influence of the size (surface effect) and strain amplitude on the fatigue damage localization (the emergence of PSBs and KBs).

Based on the results, we can deduce that the emergence of micro-KBs in combination with Polack´s model [38] probably might be the key to solving the dislocation mechanism in tongue-like extrusions. In order to have a deeper understanding of tongue-like extrusions or the evolution of the observed KBs, we analyzed an individual PSB of a larger beam (W15) in the early fatigue state with the help of the atomic force microscope (AFM) (see Figure 8b). Figure 8b clearly reveals alternating “young” micro-KBs inside the individual PSB with a characteristic distance (see arrows), which emerge perpendicular to the Burgers vector, $\underline{b}$ (see sketch in Figure 7b). That means that micro-KBs emerge after a certain cumulative strain in regions where larger deformation-induced long-range internal stresses prevail produced by the GNDs inside the PSBs. These KBs were also observed by Mughrabi in cyclic deformed Cu single crystals at a strain amplitude of about 10^−2^ [5]. Mughrabi correlated the emergence of KBs with the relaxation of long-range internal stresses. This means that at a certain higher plastic deformation, KBs develop to minimize the larger internal stresses in the microsamples. Thus, we can also deduce that the emergence of equidistant KBs correlate with the equidistant PSB walls in Figure 7a. In comparison with bulk crystals, the overall stresses are higher in microbending deformations and even much larger with decreasing sample size leading to higher internal stresses due to dislocation pile-ups and high GND densities [21,24,25,27]. Therefore, it is highly probable that the observed KBs, where the slip lines (lattice) are rotated, are responsible for the tongue-like extrusions at lower $\Delta \varepsilon_{p}$. These KBs clearly become more evident when increasing the strain amplitude or decreasing sample size.

That is also why (fewer) KBs form in the microbeams with 5 µm thickness where the role of GNDs becomes dominant in comparison to larger beams. Therefore, all larger and medium microbeams with SS orientation do not show cyclic hardening because of the local emergence of new structures (kink structures), as revealed also in the KAM maps in Figure 6 (see arrows). At this point it should be mentioned that the Bauschinger effect particularly in the W5 and W2 beams is also an additional influencing factor on the cyclic stress response (discussion below). A partially comparable tendency (no cyclic hardening) was also revealed in [25].

On the other side, cyclic hardening starts much sooner in larger beams (>2 µm) with double slip orientation (B and C orientation). Cyclic hardening in B orientation is significantly higher in comparison to the other orientations. Normally, when dislocations on different slip systems (primary and critical systems) interact, especially near the [001] orientation [46], mainly labyrinth structures form and consequently show higher cyclic hardening rates. In the case of the B orientation the critical slip system D1 is activated because of the almost same Schmid factor as the primary one A2. Consequently, dislocation locks are formed between A2 and D1 which are locally strong obstacles for dislocation movement and act as a barrier to other dislocation of the slip systems involved (latent hardening). This means, junction formations in B orientation influence the mean free path during the dislocation multiplication (Frank–Read mechanism) and thus cause a much higher cyclic hardening from the beginning. Other important dislocation interactions at the microscale, which additionally strongly influence the strength and the microstructural evolution, are discussed in [24,47]. Additionally, dislocation interactions such as Lomer–Cotrell junctions (interactions between A2 and D6) are likely to enhance the observed cyclic hardening tendency in Figure 3d. This also suggests that the activation of sessile dislocations is important for the formation of labyrinth-like structures which have an average channel width of ~0.8 ± 0.1 µm, as also observed in bulk Cu single crystals [46]. Therefore, these labyrinth-like structures can be attributed to the larger surface roughening in Figure 5. The results of larger samples can qualitatively be compared with recently published DDD simulation results by Hussein et al. [47], who investigated the effect of sample size and initial dislocation density on the dislocation structures and the corresponding cyclic mechanical response for a few cycles (<80 cycles).

### 4.2. Fatigue of Small Microbeams 

As mentioned above, the Bauschinger effect, which has a pronounced influence at the microscale [25,27], refers to the reduction of the yield stress upon reversal of loading direction after a certain amount of forward plastic deformation. The origins of the Bauschinger effect are usually related to the changes in dislocation substructures under stress reversal and in the changes in the internal stress systems [41]. During fully reversed cyclic loading under SS conditions, dislocations accumulate predominantly in the form of dislocation dipoles on primary slip systems creating positive long-range internal stresses. Long-range stresses evolve due to the interface dislocations in nonuniform microstructures (e.g., cell structures and PSBs walls), which are responsible for the accommodation of the elastic/plastic strain incompatibility between the soft and hard regions (cell interiors and cell walls or channels and PSB walls) [48]. After a strain path change, the presence of long-range stresses (back-stress) reduces the external stress magnitude required to move/degenerate polarized dislocations/structures by newly activated plastic slip leading to the Bauschinger effect [49]. An additional contribution factor to the softer reverse response during the first cycles are weak dislocation entanglements which enhance the availability of dislocation sources and dislocation mobility. This can also lead to an easier reverse flow in the material as discussed in detail by Demir et al. [27]. Therefore, we can deduce that the Bauschinger effect is responsible for the observed asymmetry between loadings in opposite directions of the microsample W5 in Figure 2a. In addition, the effect of dislocation source limitation, which increases as the sample size reduces, is the main reason for a pronounced softer reverse flow leading to stronger kinematic hardening in comparison to the larger samples with single slip orientation [24].

On the other side, cyclic hardening (the overall macroscopic RSS) in multiple slip orientations (B and C) is mostly controlled by junction formation and destruction (short range interaction) which produces larger internal stresses. These larger stresses, as well as cross-slip mechanisms are responsible for dislocation patterning [50]. The results of larger samples in Figure 3 clearly show this. While in the 5 and 2 µm beams which show softening during the first cycles, cyclic hardening begins after a certain cumulative strain when dislocation structures evolve and act as a barrier to further dislocation motion. This is the main reason for cyclic hardening through forest hardening. A similar trend was observed in DDD simulations by Hussein et al. [47,51].

However, below 2 µm a clear transition can be observed regarding the surface damaging, especially in SS orientation. Below this critical size it is highly probable that the absence of PSB-like structures, which was analytically discussed below, causes a more uniform damage deformation. That means individual dislocations on several primary and even secondary slip planes are the main reason for the not localized deformation. This is supported by the fine slip lines at the smallest beam surface (see Figure 4).

### 4.3. Influence of Length Scale on the Dislocations Inside a Slip Plane

The decrease of damage with decreasing sample size in SS orientation indicates that local plastic deformation inside extrusions caused by threading dislocations on primary slip planes is hindered in very small samples [29]. Therefore, in order to have more details about the dislocations inside a slip band we used the analytical model in (1), which is based on dislocation on parallel slip planes in thin films [29]. Thus, the extrusion width *W_ext_* correlates inversely with the saturation stress $\tau_{s}$ and is given by:

(1) $$W_{ext}=\left(\frac{\varepsilon_{pl}W}{\xi bsin\left(\lambda \right)}-1\right)\frac{µb}{8\pi \left(1-\nu \right)\tau_{s}}$$

where $\varepsilon_{pl}$ is the applied strain, *W* the sample width, ξ the number of dislocations on a single slip plane on a single cycle and λ the angle between the slip direction and the out-of-plane direction of the sample (see scheme in Figure 8b). Using Equation (1) we obtained about 22 for ξ in the larger sample with a mean *W_ext_* of 1.6 µm and around 5 dislocations in the 5 µm beam size with a mean extrusion width *W_ext_* of 4 µm (In W5 as mean *W_ext_*, the whole deformed region of the beam was measured because no PSBs such as in W15 were observed. Similarly, we obtained about 1.6 dislocations in W2 with a mean *W_ext_* of 2.6 µm). These results clearly reveal that the number of dislocations for veins formation continuously reduce when decreasing the sample size which can have a clear correlation with the fatigue extrusions, as well as with the formation of PSB walls. While in very small samples (<W2), the pronounced activated secondary systems (B5), which contribute to the formation of faint cell structures, is likely the reason for the sudden change of plastic reversibility. Additionally, the emergence of other/new dislocation mechanisms such as, e.g., vacancy diffusion or climb processes can also come into play because of the large stresses within the slip bands. In the case of multiple slip orientation, the larger and complex surface damaging can be correlated with the surface cross-slip activity, which leads to even larger surface roughness, as discussed by Hussein et al. [47,51].

### 4.4. Influence of Length Scale on the Dislocation Trapping Distance

The open question which will be addressed in this paper is why no distinct PSB dislocation structures were found in beams smaller than 5 µm. According to Brown´s bowing and passing model [52], the main contributions to the saturation stress $\tau_{s}$ are (i) the stress $\tau_{Or}$ required to bow screw dislocations in between the PSB walls and (ii) the stress $\tau_{Pass}$ required to allow screw dislocations on parallel slip planes to pass one another. The key assumption made by Brown were that $\tau_{s}$ (Equation (2)) is a linear superposition of the Orowan stress $\tau_{Or}$ and the dipole passing stress $\tau_{Pass}$, which corresponds to the annihilation of screw dislocation dipoles at high *h* which in the literature has values close to 50 nm in Cu at room temperature, while at lower temperatures DD simulations yields to values up to 16 nm [53].

(2) $$\tau_{s}=\alpha \tau_{Or}+\;\tau_{Pass}=\alpha \frac{2E_{edge}}{bd}+\frac{µb}{4\pi h}$$

In Equation (2), the coefficient α accounts for the contribution of the Orowan stress to the saturation stress (α is 0.17 in [54] and 0.5 in [52]), $E_{edge}$ is the line energy of the edge segments, *d* the channel width (In Brown´s model, the channel widths (or PSB wall spacing) are *d* = *d_c_* + *d_w_* where *d_w_* is the wall thickness and is constant with *d_w_* = 0.11 ± 0.01 µm, as well as temperature independent (*d_w_* was not measured in this work).), and *b* the Burgers vector. In bulk materials the saturation correlates with the development of PSB walls with equilibrium spacing which means that $\tau_{Or}$ has to be equal to $\tau_{Pass}$, otherwise the PSB walls are on average pulled apart or together [52]. We use this argumentation here since no measurable changes can be observed in the PSB channels when the sample size decreases (*d* remains constant, see Figure 7a). Hence, the relation between the critical dipole high *h_c_*, which corresponds to the largest dipole interaction and to a maximum $\tau_{Pass}$, and the PSB wall spacing *d* is:

(3) $$h_{c}=\frac{µb^{2}}{8\pi E_{edge}\left(2-\alpha \right)}d$$

Using Equations (2) and (3) the maximum passing stress for the critical annihilation distance *h_c_* in the PSB channels becomes:

(4) $$\tau_{c}=\;\frac{µb}{2\pi \left(2-\alpha \right)h_{c}}$$

**Figure 9:**
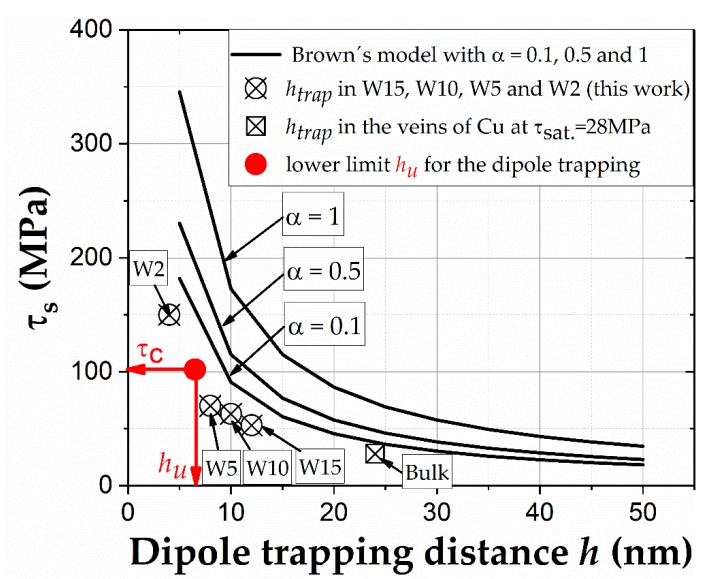
Graphical overview of the dipole trapping distance *h* of the microsamples 15, 10, 5, and 2 µm (W15, W10, W5, W2) calculated using Neumann’s Equation (5), the mean *h* of edge dislocation dipole in veins of Cu bulk single crystals, as well as the lowest critical dipole trapping distance *h_u_* with the corresponding maximal critical $\tau_{c}$. Additionally, based on Brown´s criterium (Equation (4) with α = 0.1, 0.5, and 1) the change of the trapping distance *h* for screw dislocations is also illustrated as reference.

Thus, for a given α, we observe that $\tau_{c}$ increases when *h_c_* decreases. Based on [52,54], the predictions of the bowing and passing model were found to be in reasonable agreement with experimental results in bulk materials. For example, Equation (4), with α = 0.5 [52] and 0.17 [54], yields ≈20 and 17 MPa, respectively, for the $\tau_{c}$. These values are close to the dipole passing stress of 15 MPa [54]. For the evaluation of the critical stress in Equation (4) as function of *h* we used the values for α = 0.1, 0.5, and 1 and varied *h* between 10 and 50 nm in order to cover a range of annihilation distances below the experimental value of 55 nm in bulk Cu single crystals. The results (black curves) can be found in Figure 9.

Another fundamental characteristic length at $\tau_{s}$ is the trapping distance *h_trap_* of the edge dislocations in the veins which is not considered in the above model. Based on Neumann [55], the equation for the mean trapping distance of attractive edge dipoles in slip bands *h_trap_* required for formation of dislocation microstructures is:

(5) $$h_{trap}\approx \frac{µb}{8\;\pi \left(1-\nu \right)\tau_{s}}$$

This means, when $\tau_{s}$ increases, *h_trap_* continuously decreases. For example, taking $\tau_{s}$ = 28 MPa for the bulk Cu single crystal, one obtains *h_trap_* ≈ 24 nm which agrees reasonably with mean dislocation spacing of 30 nm obtained from the dislocation density measurement. Using Equation (5) for our experiments, we obtain *h_trap_* ≈ 12, 10, 8, and 4 nm corresponding to $\tau_{s}$ ≈ 53, 63, 70, and 150 MPa for the beam sizes of 15, 10, 5, and 2 µm, respectively. All obtained results are plotted in Figure 9. The results clearly show a similar tendency such as Brown´s model if *h* of the screw dislocation in the PSB channels also decreases. These results demonstrate that the stress influences *h_trap_*. However, *h_trap_* can only reduce down to the limit *h_u_* ≥ (0.17 *R*_0_ *r_o_*)^1/2^ ≈ 6.5 nm which is associated with a maximal critical RSS $\tau_{c}$ ≈ 102 MPa using 6.5 nm in Equation (5) (*R*_0_ and *r*_0_ are the outer and inner cut-off radii, as pointed out in [16]). This lower critical value *h_u_* and the corresponding maximal stress of 102 MPa are also shown in Figure 9. Thus, we can clearly see in Figure 9 that dislocation dipoles, which constitute the veins and PSB-walls, are only to be expected with trapping distances larger than *h_u_*. This clearly explains why in our experiments only microbeams ≥ 5 µm showed PSB-like structures except smaller beams (W2) (compare experimental values in Figure 9 with BSE images in Figure 7a). This also explains that, when the sample size is further reduced (<2 µm), new dislocation mechanisms (not PSB structures) are responsible for the larger surface damaging due to the huge stresses, as mentioned above.

## 5. Conclusions

The present work investigated the effect of sample size and crystal orientation on the fatigue damage evolution and the dislocation structures in single crystalline microbeams oriented for single slip, critical and coplanar double slip. The following results were obtained:

- A clear decrease of the surface roughness in SS orientations with decreasing beam size was observed, while multiple slip orientations (B and C) showed more prevalent surface damage.
- A transition from a quasi-steady stage in the larger beams (W15) to a continuously increasing softening in the smaller beams was observed and is suggested to be mainly related to the limitation of dislocation sources in the single crystal beams.
- The emergence of micro-KBs was observed only in SS orientations in microbeams ≥ 5 µm and these disappeared around 2 µm.
- In SS orientations below 2 µm, another transition from lower to increased fatigue damage was observed (a change of the reversibility of plastic deformation). This was correlated with the marked activation of secondary systems, as well as dislocation mechanisms such as vacancy diffusion or climb processes due to the huge stresses.
- By applying Brown’s model for bowing and passing and Neumann’s equation for dislocation dipoles (Equations (4) and (5)) on our experimental results in terms of the observed PSB-like structures, we demonstrated that the characteristic length *h_trap_* of edge dislocation dipole is influenced by the stress. Thus, we could also quantitatively demonstrate the correlation of surface damage with the formation of PSB-like dislocation structures as a function of sample size and stress level in the SS orientation, as well as a critical size regime for the formation of PBS structures.

## Figures and Tables

**Table 1:** Overview of the investigated orientations A, B, C oriented for single slip (SS), critical double slip (DS), and coplanar double slip (DS) with orientation parallel to the neutral plane, thickness range of the beams, and the plastic applied strain amplitude $\Delta \varepsilon_{p}$.

Beam Axis Orientation	Oriented for	Thickness Range (µm)	$\Delta \varepsilon_{p}$
A: [-63-2]	SS	1–15	$10^{-4}-10^{-3}$
B: [-16-1]	Critical DS	1–15	$10^{-3}$
C: [5-53]	Coplanar DS	1–15	$10^{-3}$

**Table 2:** Schmid–Boas notation (S–B) for the four slip planes (SP) and slip direction (SD), as well as the corresponding Schmid factors for the orientations A, B, and C (see Table 1).

S-B	A2	A3	A6	B2	B4	B5	C1	C3	C5	D1	D4	D6
**SP, *n***	$\left(\bar{1}11\right)$			(111)			$\left(\bar{1}\bar{1}1\right)$			$\left(1\bar{1}1\right)$		
**SD, *b***	$\left[0\bar{1}1\right]$	[101]	[110]	$\left[0\bar{1}1\right]$	$\left[\bar{1}01\right]$	$\left[\bar{1}10\right]$	[011]	[101]	$\left[\bar{1}10\right]$	[011]	$\left[\bar{1}01\right]$	[110]
**A**	0.29	0.46	0.17	0.22	0.16	0.38	0.01	0.06	0.07	0.08	0.35	0.28
**B**	0.46	0.12	0.34	0.31	0.02	0.33	0.33	0.10	0.43	0.45	0.03	0.42
**C**	0.38	0.41	0.03	0.19	0.06	0.25	0.03	0.12	0.15	0.16	0.22	0.06

**Table 3:** Data of the mean resolved shear stress RSS in Figure 3 and characteristic lengths of dislocation structures in Figure 7. ***τ_ini_***: Initial RSS after the first cycle; ***τ_min._***: Minimal stress after few loading cycles; ***τ_max._***: Maximal RSS after 10^4^ cycles; ***τ_soft._*** = **|*τ_ini._*** - ***τ_min._*|**: Softening stress; ***τ_s._***: Saturation stress; *d_C_*: PSB wall spacing; *d_L_*: Labyrinth spacing; *d_trap_*: Dipole trapping distance.

	Orientation A			Orientation B			Orientation C			Bulk Material [41]
	W15	W5	W2	W15	W5	W2	W15	W5	W2	
***τ_ini._* (MPa)**	53	80	184	60	90	143	49	70	141	-
***τ_min._* (MPa)**	52	68	140	-	85	125	-	60	106	-
***τ_max._* (MPa)**	53.5	73	160	77	105	134	61	80	126	-
***τ_soft._* (MPa)**	~1	~12	~44	-	~5	~18	-	10	35	-
***τ_s_* (MPa)**	53	70	150	-	-	-	-	-	-	28
***d_C_, d_L_* (µm)**	*d_C_* ≈ 1.4±0.1			*d_L_* ≈ 0.8±0.1						*d_C_* ≈ 1.3; *d_L_* ≈ 0.8
***h_trap_* (nm)**	12	8	4	-			-			24
